# Autonomic nervous system dysfunction in schizophrenia: impact on cognitive and metabolic health

**DOI:** 10.1038/s41537-021-00151-6

**Published:** 2021-04-26

**Authors:** Nicolette Stogios, Alexander Gdanski, Philip Gerretsen, Araba F. Chintoh, Ariel Graff-Guerrero, Tarek K. Rajji, Gary Remington, Margaret K. Hahn, Sri Mahavir Agarwal

**Affiliations:** 1 Institute of Medical Science, University of Toronto, Toronto, Canada; 2 Center for Addiction and Mental Health (CAMH), Toronto, Canada; 3 Human Biology Department, University of Toronto, Toronto, Canada; 4 Department of Psychiatry, University of Toronto, Toronto, Canada

**Keywords:** Schizophrenia, Biomarkers

## Abstract

Schizophrenia (SCZ) is a psychiatric disorder characterized by a wide range of positive, negative and cognitive symptoms, along with an increased risk of metabolic syndrome and cardiovascular disease that contribute to a 15–20-year reduced life expectancy. Autonomic dysfunction, in the form of increased sympathetic activity and decreased parasympathetic activity, is postulated to be implicated in SCZ and its treatment. The aim of this narrative review is to view SCZ through an autonomic lens and synthesize the evidence relating autonomic dysfunction to different domains of SCZ. Using various methods of assessing autonomic activity, autonomic dysfunction was found to be associated with multiple aspects of SCZ pathophysiology, including symptom severity, cognitive impairment, and the development of cardiometabolic comorbidities, such as metabolic syndrome and high BMI. The strongest association of low heart rate variability was noted among patients on antipsychotic treatment with high-affinity muscarinic antagonism (i.e., clozapine, olanzapine and quetiapine). The review will also suggest ways in which studying autonomic dysfunction can help reduce morbidity and mortality associated with SCZ and its treatment.

## Introduction

The autonomic nervous system (ANS) is the branch of the peripheral nervous system that innervates organs throughout the body and is involved in the regulation of several involuntary physiologic processes, including heart rate, blood pressure and digestion, as well as emotional and behavioural regulation^1^. In 1899, Kraepelin first introduced the idea that patients with schizophrenia (SCZ) exhibit evidence of altered autonomic functioning, such as increased heart rate, sweating and salivation, and altered pupillary function, all of which suggest increased sympathetic output and decreased parasympathetic output^2–4^.

Moreover, patients with SCZ have a 15–20-year lower life expectancy than the general population, largely attributable to their almost threefold increased risk of cardiovascular disease (CVD)^5^. It is plausible that underlying autonomic dysfunction may exacerbate multi-dimensional illnesses like SCZ and predispose the individual to greater illness severity and comorbid diagnoses. Conversely, psychotic severity, chronicity of the illness, and treatment with antipsychotic (AP) medications may lead to autonomic dysfunction, thereby contributing to increased cardiometabolic risks in these patients.

Over the late 1990s and early 2000s, a number of integrative theories emerged to relate ANS functioning to behaviour. According to the Neurovisceral Integration Hypothesis, cognitive and emotional functions are regulated by brain systems also involved in the regulation of the ANS^6^. More specifically, it states that the prefrontal cortex (PFC) exerts tonic inhibition over limbic system structures that suppress parasympathetic activity and activate sympathetic activity^7^. This theory describes how autonomic, attentional and affective systems work together to form structural and functional networks that control emotion regulation and adaptive responses^6^. Similarly, Porges’ Polyvagal Theory also proposes a model of neural regulation of the ANS and provides insight on how internal physiological states relate to different types of behaviour^8^. For instance, vagal withdrawal and sympathetic dominance characterizes fight-or-flight behaviours, while vagal influence supports rest and digest and social engagement behaviours^8,9^. These theories provide potential neurobiological mechanisms linking the ANS with socio-emotional behaviours, physical illnesses and psychiatric disorders. Given this connection, psychophysiological assessment tools have been widely used to inform clinical assessments and potentially serve as an endophenotypic marker to better understand the etiology of psychological symptoms and disorders, including SCZ^10^.

In this comprehensive narrative review, we summarize the evidence relating autonomic dysfunction to the multiple domains of SCZ, including its psychopathology and associated cardiometabolic disturbance. As such, this review has three goals. First, starting with a brief overview of concepts and methods, we will discuss autonomic functioning in SCZ compared to healthy controls (HC) and between medicated and unmedicated patients. Second, we will review the association of autonomic dysfunction in SCZ with its related psychopathology and cardiometabolic comorbidities. Finally, we will discuss the therapeutic potential of monitoring and targeting autonomic dysfunction to improve functional outcomes in SCZ.

## Assessing ANS activity in SCZ

Since Kraepelin’s initial observation, an extensive literature base has developed demonstrating autonomic dysfunction in SCZ. Throughout the years, the techniques used to measure ANS activity have shifted from being invasive in nature to more non-invasive approaches (Table 1). Early quantification of ANS activity in health and disease included measuring the levels of circulating catecholamines^11^, such as norepinephrine, epinephrine and dopamine^12^, measuring electrodermal activity (EDA) responses^13^, and measuring salivary alpha-amylase (SAA) activity^14–16^. Moreover, various cardiovascular tests have been used to demonstrate parasympathetic and sympathetic reflexes. The earliest of these methods includes measuring heart rate and blood pressure changes during the process of standing up from supine position^17^. Nowadays, the most popular method of assessing autonomic functioning is through heart rate variability (HRV)^18^. This is because it is a relatively easy, accessible, and cost-effective approach that can non-invasively estimate ANS dynamics, or the balance between the sympathetic and parasympathetic nervous system branches (SNS and PSNS, respectively)^19^. In addition, it is reliable in quantifying the risk of a variety of cardiac and non-cardiac disorders, such as stroke, myocardial infarction, ischaemic heart disease, and diabetes mellitus, and can also be used to assess the autonomic effects of drugs, including psychotropic agents^20^.

**Table 1:** Methods of assessing autonomic nervous system activity.

ANS Assessment	Description & Physiological Interpretation
Plasma catecholamines	- Plasma level of circulating epinephrine, norepinephrine, dopamine^12^
	- Low sensitivity and non-specific localization of the sympathetic response^11^
Electrodermal activity (EDA)	- Skin conductance is an indicator of sweat gland activity, providing an estimate of sympathetic arousal^13^
	- ~40–60% of the schizophrenia population are EDA non-responders given their significantly suppressed EDA and skin conductance in response to noxious stimuli^41^
Salivary alpha-amylase (SAA) activity	- Salivary gland secretion (estimated through saliva sample) is regulated by both parasympathetic and sympathetic branches^14^
	- Combined with HRV analysis, increased SAA suggested to reflect sympathetic dominance and parasympathetic withdrawal^16^
Heart rate and blood pressure changes to postural position	- Moving from supine to standing position causes heart rate to increase rapidly initially and then decrease reflexively^17^
	- Standing up associated with a drop in systolic blood pressure (limited to <10 mmHg decrease due to sympathetic vasoconstriction)
Heart rate variability (HRV) analysis	- Fluctuations in time intervals between heart beats (R-R interval in continuous ECG sampling) reflect changes in autonomic regulation of the heart^21^
	- Parasympathetic activity (decreases heart rate) associated with higher HRV
	- Sympathetic activity (increases heart rate) associated with lower HRV
	- Spectral analysis of HRV provides information on the distribution of power (i.e., the variance and amplitude of the heart rhythm) as a function of frequency (i.e., the time period of the heart rhythm); separates HRV into very low frequency (VLF), low frequency (LF) and high frequency (HF) bandwidths
	- Time-domain measures are based on NN interval differences between successive QRS complexes on an ECG tracing (NN refers to normal-to-normal intervals, i.e. normal R-R intervals free from artifact)

HRV is defined as the variation in time intervals between heart beats (R-R interval in continuous ECG sampling)^21,22^. The fluctuations in these time intervals are thought to reflect changes in autonomic regulation of the heart. The ANS has control over heart rate through its modulation of the sinoatrial (SA) node^23^. Parasympathetic innervation via vagal release of acetylcholine on SA pacemaker cells slows down heart rate while sympathetic innervation increases heart rate through the release of epinephrine and norepinephrine^21^. Spectral analysis of HRV provides information on the distribution of power (i.e. the variance and amplitude of the heart rhythm) as a function of frequency (i.e. the time period of the heart rhythm). This Fast Fourier Transformation is able to separate HRV into its component very low frequency (VLF), low frequency (LF), and high frequency (HF) bandwidths^24^. These bandwidths reflect the period of time over which the rhythm occurs. Physiologic interpretations can then be made from these measurements and provide insight on health and disease (Table 2). Broadly speaking, higher HRV represents greater parasympathetic vagal innervation and is associated with flexible ANS responses to changing environmental conditions, while reduced HRV is indicative of autonomic imbalance (either a hyperactive sympathetic branch or hypoactive parasympathetic branch), and has been correlated with various pathological conditions, including CVD^25^. The HF peak is widely accepted as reflecting the efferent vagal activity of the PSNS. However, the representativeness of SNS output through HRV is somewhat controversial as the LF peak consists of both SNS and PSNS output, making it difficult to separate the dominating system^21,25^. Calculating the low to high-frequency ratio (LF/HF) ratio has been proposed as a solution to this to reflect sympathovagal balance, or to estimate sympathetic activity, though its use in the literature is also quite contentious. HRV can also be assessed through time-domain measures, which are based on NN interval differences between successive QRS complexes on an ECG tracing (NN refers to normal-to-normal intervals, i.e., normal R-R intervals free from artifact)^21^. Examples of time-domain measures include the standard deviation of all NN intervals (SDNN), which reflects both sympathetic and parasympathetic activity, and root mean square of the successive differences (RMSSD), which is a reliable estimate of vagal activity and highly correlated with HF-HRV^21^.

**Table 2:** Most common spectral and time-domain measures of HRV used in this review.

	HRV measure	Definition
Power spectral analysis measures	Total power (TP)	Represents overall ANS activity 0.03–0.4 Hz
	Very low-frequency power (VLF)	Physiological interpretation unclear ≤0.04 Hz
	Low-frequency power (LF)	Represents both SNS and PSNS activity 0.03–0.15 Hz
	High-frequency power (HF)	Represents PSNS modulation of the heart 0.15–0.4 Hz
	LF/HF ratio	Calculated to reflect sympathovagal balance or estimate sympathetic activity
Time domain measures	Standard deviation of NN intervals (SDNN)	Standard deviation of all NN Intervals
		Reflects both SNS and PSNS activity
	Root mean square of the successive differences (RMSSD)	Square root of the mean of the sum of the squares of differences between adjacent NN intervals
		Estimates vagal activity; highly correlated with HF-HRV
	Percentage of adjacent NN intervals that differ from each other by more than 50 ms (pNN50)	Number of pairs of adjacent NN intervals differing by more than 50 ms in the entire recording; divided by the total number of all NN intervals

The current review will primarily focus on characterizing autonomic dysfunction in SCZ through HRV analysis. This is because a greater amount and more recent literature exist on HRV in SCZ compared to other autonomic assessment tools. More specifically, we will focus on studies that analyzed HRV through linear algorithms (i.e., frequency and time domain measures) given their reliability in estimating ANS function^21^. Where applicable, we will also discuss findings from different autonomic function tests used in SCZ studies. A brief description of each assessment along with their physiological interpretation is provided in Tables 1 and 2.

## Characterizing autonomic dysfunction in SCZ

In comparison to non-psychiatric HCs, patients with SCZ exhibit reduced HRV and vagal cardiac control^26^. In 2016, a meta-analytic review of 34 studies quantified differences in HRV between individuals with SCZ and HCs^27^. Their findings revealed significantly lower measures of HF-HRV and RMSSD in SCZ, both of which are an estimate of efferent vagal activity of the PSNS. Subsequent meta-regressions did not show any significant effects as a function of age, illness duration, or medication status (medicated vs. unmedicated). Similarly, subgroup analyses did not reveal any differences in HRV outcomes between inpatients and outpatients. More recent studies since this review have also shown reduced HRV in SCZ patients compared to HCs and other psychiatric controls independent of medication, age or body mass index effects^28,29^. It is also noteworthy to add that autonomic dysfunction is a characteristic feature of other psychiatric illnesses, such as anxiety and mood disorders; however, it appears that autonomic dysfunction is greatest in psychotic disorders^30^.

**Fig. 1:**
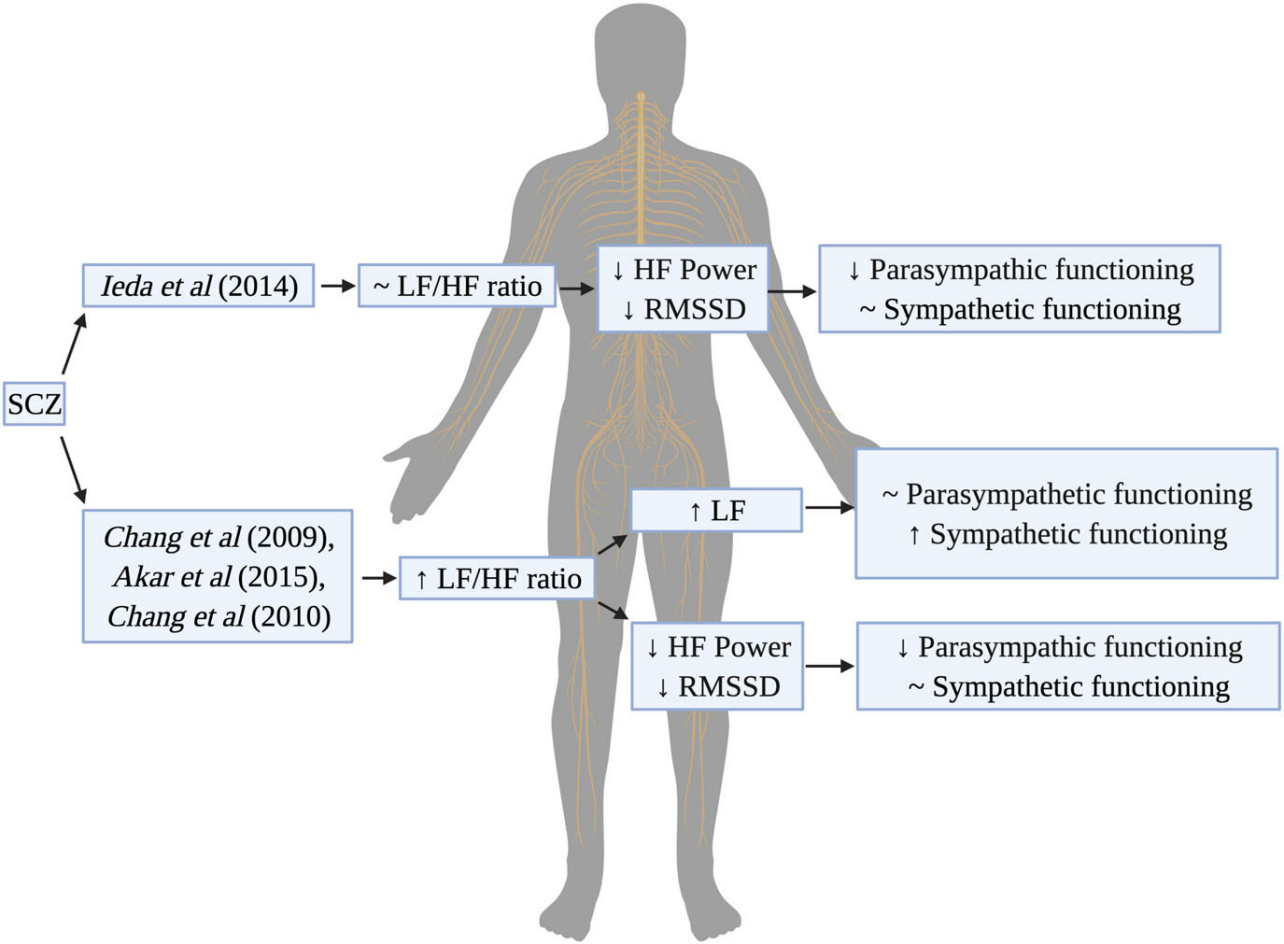
Summary of sympathovagal balance in schizophrenia. Studies of heart rate variability (HRV) use the LF/HF ratio as a proxy for estimating sympathetic activity. HRV studies in schizophrenia have found contradictory findings in terms of the LF/HF ratio. In some instances, autonomic dysfunction in SCZ can be characterized as a decreased parasympathetic functioning while maintaining relatively normal sympathetic activation (~LF/HF ratio). In other cases, the LF/HF ratio is elevated. This may represent two different scenarios: (1) increased sympathetic activation (high LF numerator) or (2) decreased parasympathetic activation (low HF denominator), leading to sympathetic dominance.

While these studies have consistently demonstrated that indicators of parasympathetic functioning, such as HF power or RMSSD, are reduced in SCZ, the question remains whether hypoactive parasympathetic function is also accompanied by a hyperactive sympathetic branch. In the study by Ieda et al.^15^, there were significant reductions in HF-HRV in SCZ compared to the HC group; however, there were no significant differences in LF/HF ratio, which can be taken as an estimate of sympathetic activity^15^. Consistent with the findings of several previous studies^31–34^, these results imply that patients with SCZ have relatively preserved sympathetic functioning and do not differ significantly with HCs. In contrast, other studies have reported a trend towards a higher LF/HF ratio in SCZ compared to HC groups^35–37^. There is some debate as to how to best interpret this finding. On the one hand, a higher value of the LF/HF ratio could imply that patients exhibit greater sympathetic modulation^26^. On the other hand, a higher ratio may just reflect a lower HF-HRV value (i.e. reduced parasympathetic/vagal outflow), suggesting that patients with SCZ preserve a relatively normal amount of SNS activity that then becomes dominant in autonomic cardiac control in the presence of impaired parasympathetic modulation (Fig. 1).

Other methods of assessing ANS activity have also demonstrated autonomic dysfunction in SCZ. For instance, in two studies that assessed SAA in SCZ, SAA activity levels were significantly higher in a group of SCZ outpatients compared to HC’s^14,15^. Within the SCZ group there were no significant differences in SAA levels between medicated and unmedicated patients^14^. Moreover, abnormal heart rate changes between the supine and standing positions have been observed in patients with SCZ (both medicated and unmedicated) compared to HCs, with heart rate elevation exceeding 30 beats per minute^38^. This is believed to be reflective of greater sympathetic activity. Lastly, results have been a bit more inconsistent in studies using EDA as an index, as individuals can be classified as either EDA responders or EDA non-responders^39^. Approximately 40–60% of the SCZ population has been classified as EDA non-responders gave their significantly suppressed skin conductance in response to noxious stimuli^40,41^; this is substantially greater than the proportion of healthy individuals that are EDA non-responders^39^.

## Autonomic dysfunction in SCZ: illness related or AP induced?

SCZ is undoubtedly associated with autonomic dysfunction. However, whether it is an intrinsic characteristic of the illness itself or the result of AP treatment has yet to be fully elucidated. This relationship can be teased out by comparing medicated versus unmedicated patients. As mentioned earlier, the meta-analysis did not find any difference in HRV markers of parasympathetic functioning between AP-naïve and medicated patients with SCZ. However, given that both first-generation and second-generation APs modulate serotonergic, dopaminergic, cholinergic and adrenergic neurotransmitter systems, it is likely that APs influence autonomic neurocardiac functioning^42^ Chang et al.^43^ conducted a case-control analysis of cardiac autonomic dysfunction in a group of 314 unmedicated patients with acute SCZ and 409 HCs. Frequency domain indices of HRV revealed that unmedicated patients had consistently faster mean heart rates and reduced HF-HRV levels compared to HCs. Similarly, another study examining HRV in acute first-episode drug naïve psychotic patients also found reduced RMSSD, indicating decreased variation in consecutive heartbeats, as well as significantly less HF power during psychosis compared to HCs^44^. Elsewhere, another study did not show any significant differences in the amount of HRV reduction exhibited by a group of medicated and unmedicated first-episode psychosis patients^29^. These findings imply that impaired neurocardiac regulation may be related to the illness itself and not secondary to the chronicity of the illness or AP treatment. Low resting-state vagal modulation/parasympathetic activity is also observed in a variety of other psychiatric conditions, such as mood disorders, anxiety disorders and autism spectrum disorder^30,45^. To this point, many scholars have suggested that reduced HRV may be a transdiagnostic factor associated with baseline psychological discomfort, reduced mental flexibility, stress, and other behavioural factors^46,47^.

## Effects of APs on ANS activity

**Fig. 2:**
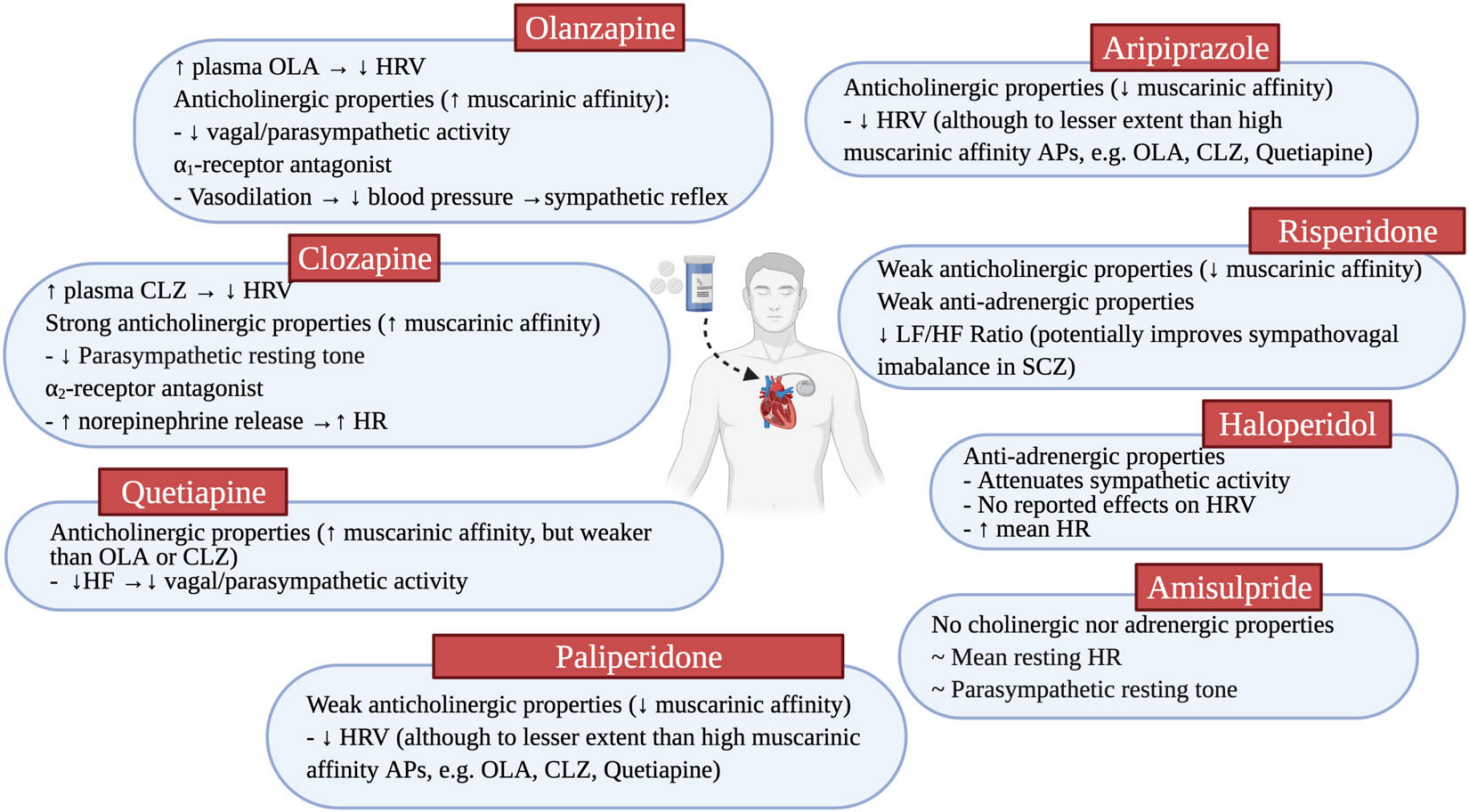
Antipsychotic properties and their effects on autonomic functioning and heart rate variability. The differing effects of antipsychotics (AP) on autonomic dysfunction and heart rate variability (HRV) may be dependent on the pharmacokinetic properties of the APs (i.e. their affinities for cholinergic and adrenergic receptors).

The effects of APs on autonomic functioning and sympathovagal balance remain elusive^26^. The extent to which APs exacerbate autonomic dysfunction in SCZ may be dependent on the type of AP taken and their respective mechanisms of action in the body (i.e., interaction with different neurotransmitter systems/receptors) (Fig. 2)^33^. There appears to be a negative correlation between neurocardiac control and the degree of AP affinity and antagonism of muscarinic receptors (M1-M5), with high muscarinic affinity APs (clozapine, olanzapine and quetiapine) showing greater reductions in HRV than low muscarinic affinity APs (risperidone and aripiprazole)^48–50^. Amisulpride does not have any cholinergic or adrenergic properties and has no significant effects on autonomic function^42^. In the cases of olanzapine and clozapine, antiadrenergic properties also play a role in their modulation of the ANS. For example, olanzapine blockade of α_1_-adrenergic receptors causes vasodilation and reduction in blood pressure, which thereby initiates a reflexive sympathetic response. Similarly, clozapine enhances noradrenergic activity through its blockade of α_2_ receptors, which are presynaptic modulators of norepinephrine release; thus blocking these receptors allow for increased release of norepinephrine^51^. Indeed, studies examining catecholamine levels have revealed that clozapine increases plasma norepinephrine by almost sixfold^52^, in contrast to fluphenazine^53^ and haloperidol^52^ which were not shown to cause any significant spillover of norepinephrine.

Furthermore, the exacerbation of autonomic dysfunction with APs has been found to be dose dependent, with multiple regression analyses revealing statistically significant associations between daily dose of AP drugs and PSNS activity^33^. More specifically, significant decreases in HRV and ANS activity were only observed with AP doses ≥501 mg/day chlorpromazine equivalent. In addition, it should be noted that this study did not find any effects of anticholinergic antiparkinsonian agents on ANS activity; however, given the low dose of these agents in the study (3.2 ± 1.5 mg/day biperiden equivalent), the effects of such agents may not only be related to their anticholinergic affinity but also dose. Lastly, the effects of APs on ANS activity may also be related to their route of administration as one study to date has provided evidence that long-acting injectable APs have fewer adverse effects on ANS activity, and particularly SNS activity (as assessed through LF-HRV), in comparison to oral APs^54^. This finding is likely due to the different pharmacokinetic profiles of these drug formulations given that a single injection once or twice a month provides a more steady state of the AP in the blood than daily oral dosing would^55^. Significant positive associations have been made between plasma concentration and adverse events of APs^56^; therefore, long-acting injectables warrant further investigation to determine if they may curb autonomic effects of APs and potentially limit the occurrence of adverse side effects of the medications.

## Implications of autonomic dysfunction in SCZ

### Psychiatric symptom severity

Autonomic dysfunction in SCZ may be implicated in the pathophysiology of its primary positive and negative symptoms. Suppressed vagal modulation in SCZ is thought to be a consequence of long-lasting stressful experiences associated with the psychotic state^57^. Several studies have found a significant negative correlation with the Positive and Negative Syndrome Scale (PANSS) total score and time-domain measures SDNN and RMSSD^58^, as well as other HRV parameters of PSNS activity^57,59,60^. This implies that greater symptom severity (indicated by a higher PANSS score) is associated with poorer autonomic functioning^57–60^. It should also be noted that these associations remained significant when controlling for covariates such as age, sex, BMI, drug and nicotine use, and anticholinergic medication status^60^. Moreover, factor analysis of a five-factor model of the PANSS revealed negative correlations between SDNN and RMSSD and the cognitive/disorganization factor, suggesting a correlation between autonomic function and this symptom cluster^58^. Elsewhere, the negative symptom domain of the PANSS, which focuses largely on distortions in emotional, social and thinking processes, was inversely related to parasympathetic HRV parameters RMSSD^34^ and HF^28,34,61^ and positively associated with the LF/HF ratio^34^; the apathy and withdrawal scales of the PANSS have also been negatively correlated with PSNS HRV indices^44^. Only one study was identified that provided contradictory results, indicating no association between HRV and any of the symptom domains of SCZ, as assessed by the PANSS^31^.

Other scales of assessing symptom severity have also been correlated with autonomic functioning. For instance, higher scores on the Brief Psychiatric Rating Scale (BPRS) have been correlated with lower HF-HRV and greater LF/HF ratios^62^, indicating that patients with stronger psychotic symptoms exhibit reduced cardiovagal modulation. However, while Henry et al.^32^ failed to note any correlations between HRV and BPRS scores among a group of SCZ patients, they did find a significant negative correlation between HF-HRV as well as a significant positive correlation with the total score on the Young Mania Rating Score (YMRS). Moreover, higher BPRS scores among SCZ patients have also been correlated with higher SAA levels^14,15^, as well as higher EDA levels during rest and in response to novel innocuous stimuli^63,64^. Interestingly, different patterns of symptomology have been noted in EDA responders versus non-responders. EDA responders exhibit greater aggressive, manic and anxious symptoms^65^ in comparison to non-responders who tend to score higher on BPRS rating scales of emotional withdrawal, depressed mood and flattened affect^66^. Furthermore, patients with lower scores on the Global Assessment Functioning (GAF) scale exhibit lower overall ANS functioning and lower parasympathetic outflow in comparison to patients with higher GAF scores^19^, even when controlling for age, gender, body mass index, AP dose, and lipid profiles. In contrast, one study that employed the Scale for the Assessment of Positive Symptoms (SAPS) and the Scale for the Assessment of Negative Symptoms (SANS) found no significant correlations between HF-HRV and symptom severity in both unmedicated early psychosis patients and those treated with anticholinergic agents^29^.

Despite some contradictory evidence, there appears to be consensus that symptom severity is inversely related to HRV indices of vagal modulation and positively correlated with other indicators of sympathetic arousal (see summary in Supplementary Table 1). This supports the well-established notion that exposure of remitted patients to stressful life events or relatives with high expressed emotion makes them susceptible to earlier relapse^67^, as well as the finding that elevated autonomic activity tends to precede psychotic episodes^68^. It is postulated that the relationship with HRV may be mediated by the reduced activity in the amygdala–prefrontal circuits that have been described in SCZ^69^. These neural structures are implicated in the coordination of cardiovagal modulation, particularly in the presence of an arousing or potentially threatening stimulus. Under normal conditions, this circuit is involved in appraising the situation to coordinate an appropriate autonomic response and maintain homeostasis. In a group of paranoid SCZ patients (DSM IV criteria), with persistently high arousal states, the lack of engagement between these brain regions may lead to preservation and exacerbation of arousal responses and a shift in sympathovagal balance influencing the heart^28,69^. This in turn can intensify symptoms of hypervigilance and paranoia^69^.

### Cognition

Another core domain of SCZ pathophysiology includes impairments in mental processes such as attention, memory, perception, executive functioning and social cognition^70^. These impairments are highly correlated with the degree of impairment in social, occupational and adaptive functioning in patients^71^. As such, teasing out the relationship between autonomic function and cognitive processes may provide some insight on the development and maintenance of cognitive impairments in psychiatric illnesses.

Among SCZ patients, studies have shown evidence of a relationship between cognitive functioning and autonomic activity (see Supplementary Table 2). In an EDA study that administered an extensive neurocognitive test battery, EDA non-responders displayed difficulties in verbal comprehension/fluency, short and long-term memory, perceptual organization, psychomotor function, abstract reasoning and executive function^72^. EDA responders consistently had intermediate scores on executive function tests, performing worse than controls but better than EDA non-responder patients. These findings, however, contradict an earlier study that found no significant difference in performance on the MATRICS battery between EDA responding and non-responding patients, and a trend approaching significance for better results among EDA non-responders^73^.

**Fig. 3:**
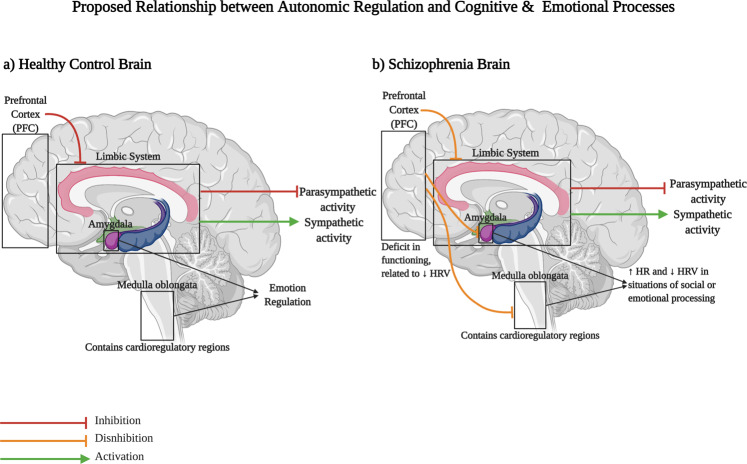
The Neurovisceral Integration Hypothesis. **a** Cognitive and emotion processing functions are regulated by brain systems also involved in the regulation of the autonomic nervous system. The Neurovisceral Integration Hypothesis asserts that the prefrontal cortex (PFC) exerts tonic inhibition over limbic brain regions (curved red line) that typically suppress parasympathetic responses (straight red line) and activate sympathetic responses (straight green arrow). **b** In schizophrenia, deficits in the PFC may lead to disinhibition of the amygdala and medullary regions (orange lines) during situations of emotional or social processing. This results in suppression of parasympathetic activity and stimulation of sympathetic activity, thereby causing increased heart rate and decreased heart rate variability. This may potentially be an explanatory mechanism underlying the correlation between decreased HRV and reduced PFC activity.

Interestingly, a recent review of non-psychiatric patients presented evidence that, under resting conditions, HF-HRV is associated with greater activity in brain regions involved in working memory, executive functioning, decision making and socially driven interactions, including the right dorsolateral PFC (DLPFC), right superior frontal cortex, and anterior cingulate cortex^74^. Moreover, HRV parameters have been positively correlated with performance on tests of global cognitive functioning, processing speed and working memory^75^, and reduced HRV has been independently associated with abnormal PFC activity^76^ and reduced regional cerebral blood flow^77^ in healthy individuals. While no studies to date have investigated HRV in relation to mental processes such as attention and working memory in SCZ, there have been studies focused on the role of autonomic function in social cognition (see Supplementary Table 2). Reduced HRV has been consistently reported in studies where individuals are exposed to negative social situations^78^, indicating HRV is a reliable marker of social cognitive processes^79^. This finding has also been replicated in SCZ studies. For instance, Jauregui et al.^80^ found that patients with SCZ displayed abnormal decreases in both HF-HRV and LF-HRV in response to a variety of social cognition tasks, as compared to HCs and unaffected first degree relatives. Similarly, in a recent 2019 study, social functioning in SCZ was negatively associated with the LF/HF ratio, which represents less flexibility in autonomic functioning^81^. Per the theory of neurovisceral integration^6^, deficits in PFC functioning, which has been related to reduced HRV, may lead to disinhibition of the amygdala and cardioregulatory regions in the medulla, thereby leading to increased heart rate and decreased HRV in situations of social or emotion processing (Fig. 3). On the other hand, Porges’ polyvagal theory asserts that autonomic balance and the inhibitory influence of the vagus nerve on the SA node create a psychophysiological state that encourages social engagement and prosocial behaviours^9^. Although these two theories postulate differing mechanisms, both allude to the idea that efficient cardiac control allows for greater adaptability to changing environmental demands and better emotion regulation^82^. This implies that reductions in HRV may be associated with social impairment in SCZ, which consequently may contribute to illness maintenance as social disengagement makes treatment and recovery difficult^82^.

Furthermore, given the importance of cholinergic neurotransmission in attentional orientation, it can be expected that disruptions to the cholinergic system may have adverse impacts on cognitive functioning^83^. AP treatment often exposes patients to a high anticholinergic burden (ACB) through competitive inhibition of muscarinic receptors and the subsequent acetylcholine-mediated response^84^. In a cross-sectional analysis of 233 community-dwelling participants with SCZ or schizoaffective disorder on APs, it was found that 63.7% of participants had severe ACB, and that ACB scores were negatively associated with functional capacity and cognitive domains, such as attention/vigilance and speed of processing^85^. Subgroup analysis revealed that the effect of ACB on cognition and function was greatest among patients 55 years or older. These results indicate that, despite their therapeutic benefits, the anticholinergic properties of APs may aggravate autonomic imbalance and exacerbate cognitive impairments in SCZ. Further research is warranted on the use of autonomic function tests as a way of assessing the amount of ACB patients experience and developing an effective treatment to mitigate its effects and thereby improve the patient’s cognitive abilities and overall functional capacity.

### Cardiometabolic comorbidities

Patients with SCZ have a 20% lower life expectancy than the general population owing to their 2–3 fold increased risk of CVD^5^. Key factors in this include a constellation of metabolic aberrations that constitute metabolic syndrome, including obesity, dyslipidemia, insulin resistance and diabetes, and hypertension. HRV is the most popular assessment of autonomic activity that has been related to general and cardiovascular health. Indeed, lower HRV has been independently associated with individual CVD risk factors, including hypertension^86^, diabetes^87^ and high cholesterol^88^ in non-psychiatric populations. These findings have also been replicated in a group of SCZ patients (see Supplementary Table 3)^34,89^. However, a large correlational study failed to note any significant differences in HRV between SCZ patients with comorbid metabolic syndrome and those without^90^. This is in contrast to the pattern that was observed in the HC group, whereby HCs with metabolic syndrome exhibited lower HRV than HC’s without. Nonetheless, reduced HRV is considered a reliable early biomarker for CVD and all-cause mortality^1,91^.

Given the largely cross-sectional nature of the reporting literature, it is difficult to determine causality and discern whether metabolic aberrations are the cause or consequence of autonomic dysfunction. On the one hand, autonomic neuropathy and autonomic imbalance are well-reported complications of diabetes and have growing evidence of resulting from obesity and metabolic syndrome as well^92^. In these disease states, it is believed that the balance between the two autonomic branches shifts towards SNS predominance. In the case of diabetes, this SNS predominance is speculated to be a downstream effect of hyperinsulinemia, hyperglycaemia and insulin resistance^92^, while obesity may trigger increased SNS activity as an adaptive mechanism to increase resting energy expenditure and compensate for the positive energy balance^93^. Furthermore, lifestyle interventions such as exercise and eating a healthy diet have been shown to be effective in reducing SNS activation, which in part may be mediated by improving these cardiometabolic risk factors^92,94^.

Conversely, the inverse relationship also exists in that these metabolic changes may be partially induced by a shift in sympathovagal balance towards sympathetic hyperactivity. Increased sympathetic outflow leads to suppressed insulin release and high levels of circulating epinephrine, norepinephrine, glucagon and cortisol, all of which promote glycogenolysis and endogenous glucose production, thereby inducing hyperglycaemia^95^. These neuroendocrine factors also stimulate adipose lipolysis which releases free fatty acids into the circulation and leads to dyslipidemia^96^. Unsurprisingly, excessive sympathetic activation has detrimental effects on the cardiovascular system, contributing to the development of cardiovascular hypertrophy, arrhythmias, and hypertension^97^. In SCZ, APs add significantly to this risk of metabolic dysregulation, with clozapine and olanzapine carrying the greatest metabolic liability^98,99^. APs influence autonomic functioning by binding and inhibiting dopamine D2, α-adrenergic and cholinergic M3 neurotransmitter receptors in the central and peripheral nervous system which, synergistically, may induce these cardiometabolic side-effects^100,101^. Scigliano et al.^101^ have proposed a pathogenic hypothesis that centres around autonomic dysfunction due to chronic D2 receptor blockade by APs. D2 receptors modulate the release of epinephrine and norepinephrine from sympathetic terminals; abolishing peripheral dopaminergic modulation results in increased sympathetic tone through excessive stimulation of α-adrenergic receptors. Simultaneous inhibition of muscarinic receptors then prevents a reflexive parasympathetic response to maintain autonomic balance. Impaired glycemic control, dyslipidemia, hypertension, and increased risk of cardiac arrhythmias are some of the medical sequalae of this chronic sympathetic activation.

Lastly, the vagus nerve is a chief mediator of the bidirectional communication along the gut-brain axis through cholinergic activation of nicotinic receptors^102^. The ANS regulates gut functions including regional motility, secretion, permeability and mucosal immune response and can induce changes in gut microbiome (GMB) composition and activity^103^. In turn, sensory afferent neurons of the vagus nerve detect a diverse range of chemical and mechanical stimuli within the intestines and GMB, and transmit messages to the nucleus tractus solitarius in the brainstem to initiate autonomic, endocrine and behavioural responses^104^. A systematic review has revealed that patients with psychotic, bipolar and depressive disorders display altered abundances of microbial organisms in comparison to HCs^105^. Moreover, APs are believed to induce or exacerbate GMB changes^106^. Thus, it is plausible that alterations in the functioning of the GMB in SCZ may cause aberrant vagal signalling that can lead to cardiometabolic disturbances. Further research is warranted to tease out the relationship between autonomic dysfunction and microbial dysbiosis in SCZ.

## Clinical and research implications

**Fig. 4:**
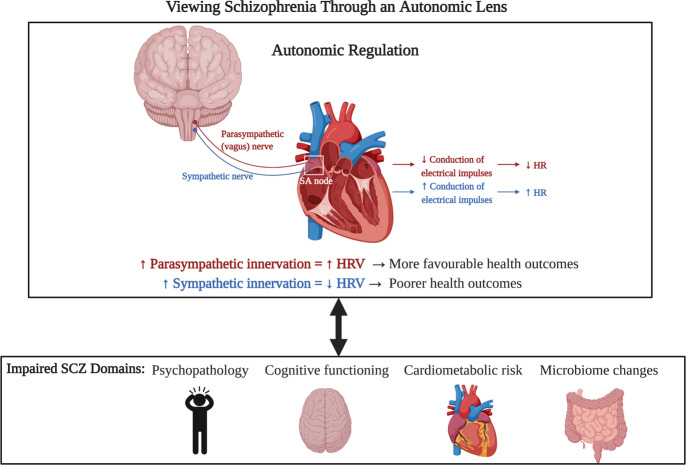
Implications of autonomic nervous system dysfunction in schizophrenia. Autonomic dysfunction, in the form of increased sympathetic activity and decreased parasympathetic activity, has been implicated in various disease states. Autonomic dysfunction, as assessed through heart rate variability (HRV) and other assessment methods, has been related to various domains of SCZ, including psychopathology, cognitive functioning, cardiometabolic risk, and microbiome changes. The relationship between these domains and autonomic functioning is believed to be bidirectional. SCZ Schizophrenia, HR heart rate, HRV heart rate variability.

Recognizing autonomic dysfunction could be associated with diverse physical, mental, and/or behavioural symptoms in SCZ is of great importance as all these domains contribute to poor medication compliance, worsened quality of life, and increased mortality rate (Fig. 4)^19,101^. Further research using simple, non-invasive and reliable measures of autonomic functioning may allow for identifying impending relapse or subclinical signs of AP-induced cardiometabolic side effects and worsening psychopathology or cognitive dysfunction. For example, Huang et al.^61^ have suggested that the use of HRV may be an applicable biomarker for treatment response on negative symptoms, given the well-reported inverse association between HRV and negative symptom severity, as well as a screening tool for cardiovascular risk stratification and early intervention. Targeting autonomic dysfunction also opens up a new avenue for therapeutic intervention to enhance psychophysiological functioning and improve functional outcomes in SCZ. For example, in one randomized controlled trial of short 20-min HRV-biofeedback for psychotic symptoms, participants with subclinical symptoms were trained in diaphragmatic breathing and taught about its application for increasing HRV^107^. When the proper protocol was followed, the biofeedback group exhibited a larger increase in overall HRV (while no change in the mainly vagal HRV component, RMSSD) than active and waiting controls and also led to improvements in perceived control and state paranoia. This indicates that brief biofeedback sessions have the potential to induce short-term benefit; however, longer interventions are required to properly engage breathing and stimulate the baroreflex. In another study, individuals at high risk for psychosis that underwent a 4-week HRV-biofeedback intervention showed a significant decrease in impaired ability to tolerate normal stressors and dysphoric mood^108^. It would be worthwhile to explore the efficacy of biofeedback in a clinical SCZ population. Mindfulness based cognitive-behavioural therapy is also another potential intervention, and has been found to attenuate reduction in HF-HRV and improve emotional processing in bipolar disorder^109^. A more invasive intervention that has been shown to increase HRV and shift autonomic cardiac control towards parasympathetic predominance in HCs is vagal nerve stimulation (VNS), which involves implanting electrodes on the vagus nerve and using electrical pulses to generate firing potentials^110^. VNS has been approved for the treatment of epilepsy and treatment-refractory depression^110^ and has shown initial evidence for weight loss in these two populations^111^. To date, only one small pilot study has employed VNS in SCZ and showed the intervention was well tolerated with an overall trend towards improved SCZ symptoms; however, compliance was low which questions the feasibility of a patient-controlled neurostimulation intervention^112^. Further investigation may be warranted to understand the impact of VNS on autonomic functioning in SCZ in relation to symptom severity as well as cardiometabolic disturbances.

## Conclusion

To summarize, evidence of autonomic dysfunction in SCZ, whether related to the illness itself or secondary to AP effects, suggests that patients have an imbalance in parasympathetic and sympathetic functioning and in turn have greater difficulty adapting to changing environmental demands. This shift in equilibrium may be reflected in various domains of the illness (Fig. 4). Targeting ANS activity may help reduce morbidity and mortality associated with SCZ and its treatment. This may also encourage novel therapeutic strategies to improve outcomes in SCZ.

### Reporting summary

Further information on research design is available in the Nature Research Reporting Summary linked to this article.

## Supplementary information

Supplementary Information

Reporting Summary
