# Supplementary material for: Autonomic nervous system dysfunction in schizophrenia: impact on cognitive and metabolic health
Source: NPJ Schizophr. 2021 Apr 26;7:22. doi: 10.1038/s41537-021-00151-6 (PMC8076312; doi:10.1038/s41537-021-00151-6)
Supplement: Supplementary file 1 — Supplementary Information [file 41537_2021_151_MOESM1_ESM.pdf]

Supplementary Tables

Supplementary Table 1: Summary of studies assessing autonomic dysfunction in relation to SCZ symptom severity

Author & Year	N	Sex (% Male)	Mean Age (SD)	Index of Autonomic Function	Symptom Severity Scales	Significant Outcomes
Heart Rate Variability Studies						
Kim et al (2011)	SCZ=21<br>HC=21	52% M<br>(both groups)	SCZ: 33.0<br>(11.5)	SDNN<br>RMSSD<br>LF/HF Ratio	PANSS	- Severity of psychotic symptoms associated with reduced HRV<br>- Cognitive/disorganization factor negatively correlated with SDNN and RMSSD
Quintana et al (2016)	SCZ=47<br>BD=33<br>HC=21	SCZ: 73%M<br>BD: 45% M<br>HC: 57% M	SCZ:29.8 (8.4)<br>BD: 30.9 (10.6)<br>HC: 31.7 (7.7)	IBI (interbeat interval)<br>HF	PANSS	- SZ and BD are associated with reduced HRV in comparison with controls.<br>- Patients with reduced HRV had increased overall and negative psychosis symptom severity
Chung et al (2013)	SCZ=94<br>HC=51	SCZ: 88% M<br>HC: 82.3% M	SCZ: 40.8 (8.9)<br>HC: 41.1 (9.1)	SDNN<br>RMSSD<br>pNN50<br>HF<br>LF<br>VLF<br>LF/HF Ratio	PANSS<br>GAF	- SDNN and vagal-related HRV indices (RMSSD, pNN50, HF power) were negatively correlated with severity of psychopathology<br>- LF/HF ratio (sympathovagal balance) was positively correlated with the negative scale of the PANSS and GAF
Valkonen-Korhonen et al (2003)	FEP=18<br>HC=21	FEP: 44% M<br>HC: 23.8% M	FEP: 27 (12.3)<br>HC: 29 (7.3)	RRI (RR-intervals)<br>RMSSD<br>pNN50<br>HF<br>LF	PANSS	- On the PANSS scores, apathy, social withdrawal, and invert withdrawal were correlated with several HRV parameters and HR reactivity

Mujica-Parodi et al (2005)	SCZ=19<br>HC=24	SCZ: 58% M<br>HC: 46% M	SCZ: 34.7 (10.3)<br>HC: 34 (8.1)	LF<br>HF	PANSS	- No association between HRV and any of the symptom domains of SCZ on the PANSS
Henry et al (2010)	SCZ=14<br>BD=23<br>HC=23	SCZ: 64% M<br>BD: 61% M<br>HC: 57% M	SCZ: 32.9 (2.9)<br>BD: 32.9 (2.6)<br>HC: 29.4 (2.0)	SDNN<br>RMSSD<br>pNN50<br>LF<br>HF<br>LF/HF Ratio	BPRS<br>YMRS	- Total YMRS score was significantly correlated with a higher LF/HF ratio in all patients and was negatively correlated with HF power in the SCZ group.<br>- There was a negative correlation between HF power and BPRS scores in the BD group, but no correlations with any HRV measures and BPRS in SCZ patients.
Bar et al (2008)	SCZ=40<br>HC=58	SCZ: 73% M<br>HC: 67% M	SCZ: 34.7 (7.1)<br>HC: 33.8 (8.4)	RMSSD<br>LF<br>HF	BPRS	- Patients displaying stronger psychotic symptoms according to the BPRS total score exhibit more severe cardiac autonomic disturbances, as indicated by reduced HF power and increased LF/HF ratio
Fujibayashi et al (2009)	SCZ=71<br>HC=72	SCZ: 32% M<br>HC: 41 % M	SCZ: 56.2 (1.7)<br>HC: 52.2 (1.2)	LF<br>HF<br>TP	GAF	- Overall ANS (TP) and parasympathetic nervous system activity (HF) were significantly reduced in the low-GAF as compared to the high-GAF group.
Cacciotti-Saija et al (2018)	FEP=48<br>HC=48	SCZ: 71% M<br>HC: 71% M	SCZ: 21.9 (4.3)<br>HC: 22.0 (4.7)	HF<br>RMSSD	SAPS<br>SANS	- No significant correlations between HF or RMSSD and symptom severity in both unmedicated early psychosis patients and those treated with anticholinergic agents.

Huang et al (2020)	SCZ=63	SCZ: 48% M	SCZ: 46.7 (9.0)	Mean HR<br>SDNN<br>TP<br>VLF<br>LF<br>HF<br>LF/HF Ratio	PANSS<br>PSP	- Negative correlations between the PANSS negative score and TP, LF, and HF.<br>- Negative association between negative symptom severity and parasympathetic activity was significant.<br>- BMI was negatively related with HF<br>- Use of high muscarinic affinity AP was negatively associated with LF
Benjamin et al (2020)	SCZ=35<br>BD=52<br>HC=149	SCZ: 46% M<br>BD: 40% M<br>HC: 49% M	SCZ: 32.8 (11.0)<br>BD: 33.9 (12.1)<br>HC: 36.4 (10.3)	HF-HRV	PANSS<br>GAF<br>YMRS	- HRV was significantly lower in both clinical groups compared to HCs; no significant HRV differences between patient groups.<br>- PANSS total score was significantly associated with HRV<br>- Positive correlations were found between HRV and GAF symptom and function scores<br>- Significant negative correlations between YRMS score and HRV<br>- Covariates included: age, BMI, anticholinergic medication status, sex, drug and nicotine use.
Salivary Alpha Amylase Studies						
Inagaki et al 2010	SCZ=54<br>HC=55	SCZ: 43% M<br>HC: 51% M	SCZ: 44.15 (11.35)<br>HC: 41.06 (11.01)	sAA	BPRS	- sAA was found to be larger in SCZ patients than HCs<br>- Strong correlation between sAA and psychiatric symptoms<br>- In SCZ patients, larger SNS functioning over PSNS functioning may be involved in the increased amounts of sAA

Ieda et al 2014	SCZ=25<br>HC=25	SCZ: 48% M<br>HC: 44% M	SCZ: 48.16 (12.39)<br>HC: 45.04 (13.62)	sAA	BPRS	- SCZ patients were found to have larger amounts of sAA than HCs<br>- SCZ patients were found to have lesser PSNS functioning (reduced HRV) than HCs<br>SCZ patients and HCs did not significantly differ in SNS functioning
Zahn et al 2005	SCZ=73	SCZ: 70% M	SCZ: 31.2 (7.6)	sAA	BPRS	Compared to SCZ patients with lesser electrodermal responsivity and autonomic baselines, SCZ patients with larger electrodermal responsivity and autonomic baselines had greater amounts of symptoms
Electrodermal Activity Studies						
Gruzelier et al 1976	Non-institutionalized patients = 30<br><br>Institutionalized patients = 30	SCZ: 100% M	Non-institutionalized responders: 35.47 (10.02)<br><br>Non-institutionalized non-responders: 36.93 (10.63)<br><br>Institutionalized responders: 47.5 (8.0)<br><br>Institutionalized non-responders: 47.7 (7.4)	EDA	Wittenborn Psychiatric Rating Scale	- Non-institutionalized patients: responders were more highly aroused psychophysiologically and were rated as more anxious, manic, assaultive and attention demanding than non-responders.<br>- Institutionalized patients: skin conductance levels and spontaneous fluctuations were associated with behavioural<br>- Amongst institutionalized patients, skin conductance levels and spontaneous fluctuations were associated with behaviour of a schizophrenic character.<br>Overall, EDA responses of SCZ patients at rest are related to facts of clinical behaviour.

Bernstein et al 1981	SCZ=40<br><br>Non-SCZ=40<br><br>HC=40	SCZ=63% M<br><br>Non-SCZ=50% M<br><br>HC=60% M	SCZ= 32.3 (9.2)<br><br>Non-SCZ=31.7 (8.9)<br><br>HC=32.3 (9.2)	EDA	BPRS	SCZ non-responders showed significantly greater emotional withdrawal and conceptual disorganization; had a tendency toward more blunted affect and significantly less excitement than did responder schizophrenics
Schell et al 2005	SCZ=78<br>HC=36	SCZ=85% M<br>HC=72% M	SCZ=23.4 (4.3)<br>HC=24.4 (3.8)	EDA	BPRS factor score of Anergia<br><br>SANS	- Higher tonic electrodermal arousal were associated with poorer functional outcome and greater negative symptoms at 1-year follow-up.<br>- Skin conductance was positively correlated with the BPRS anergia factor score and SANS score<br>- Skin conductance orienting responders had higher BPRS Anergia and SANS scores than non-responders

SCZ = Schizophrenia, **HC** = Healthy Controls, **BD** = Bipolar Disorder, **FEP** = First episode psychosis, **HR** = Heart Rate, **LF** = Low frequency power, **HF** = High frequency power, **TP** = Total Power, **RMSSD** = Root Mean Square of the Successive Differences, **SDNN** = Standard Deviation of NN Intervals, **pNN50** = Mean number of times an hour in which the change in successive normal sinus (NN) intervals exceeds 50 ms, **SAA =** Salivary Alpha Amylase, **EDA =** Electrodermal Activity, **PANSS**= Positive and Negative Syndrome Scale, **GAF** = Global Assessment of Functioning Scale, **BPRS** = Brief Psychiatric Rating Scale, **YMRS** = Young Mania Rating Score, **SAPS** = Scale for the Assessment of Positive Symptoms, **SANS** = Scale for the Assessment of Negative Symptoms, **PSP** = Personal and Social Performance Scale, **BMI** = Body Mass Index, **AP** = Antipsychotic

Supplementary Table 2: Summary of studies assessing autonomic dysfunction in relation to cognitive functioning in SCZ

Author & Year	N	Sex (% Male)	Mean Age (SD)	Index of Autonomic Function	Cognitive Assessment	Significant Outcomes
Heart Rate Variability Studies						
Jauregui et al (2011)	SCZ=19<br>FDR=19<br>HC=19	SCZ:71%<br>FDR:47%<br>HC:58%	SCZ:29.7 (9.2)<br>FDR: 51 (18.8)<br>HC:27.6 (6.0)	RRI<br>SDNN<br>LF<br>HF	- Facial Recognition Test<br>- Baron-Cohen Faces Test<br>- Baron-Cohen Reading the Mind in the Eyes Test<br>- The Faux Pas Test<br>- Theory of Mind Tests	- Social cognition tasks induced a shortening of the RR interval in unaffected relatives, but not in patients<br>- Decrease in HF and LF-HRV in patients during social cognition tasks<br>- LF-HRV higher in patients during a theory of mind task than a control task
Kim et al (2019)	SCZ=27<br>BD=30<br>HC=25	SCZ:40.7%<br>BD:33.3%<br>HC:44.0%	SCZ:42.5 (11.9)<br>BD: 39.7 (12.61)<br>HC: 42.5 (12.6)	LF<br>HF<br>LF/HF	- Questionnaire of social functioning<br>- Basic Empathy Scale<br>- Social Attribution Task-Multiple Choice	- In SCZ, social functioning was negatively associated with LF/HF ratio indicated higher SNS activity in comparison to PSNS activity which may result in less flexible ANS<br>- In BD, social functioning was significantly associated with HF; reduced HF implies a difficulty in regulating one's emotional arousal.
Electrodermal Activity Studies						
Nilsson et al 2015	SCZ=25<br>HC=14	SCZ=72% M<br>HC=64% M	SCZ=32.0 (8.7)<br>HC=33.3 (8.4)	EDA	- Wechsler Adult Intelligence Scales (WAIS)<br>- The Rey Auditory Verbal Learning test	- Overall lower performance on an extensive neurocognitive test battery in EDA non-responding patients with SCZ compared to HC

					- The Controlled Oral Word Association Test<br>- Fingertapping Test<br>- The Trail Making Tests<br>- The Wisconsin Card Sorting Test<br>- Benton Visual Retention Test	- EDA non-responders exhibited difficulties on all tests, with the greatest difference being in the WAIS digit symbol test.<br>- EDA responders had intermediate scores (higher than EDA non-responders but consistently lower than HCs).
Ikezawa et al 2012	SCZ=28<br>HC=24	SCZ=54% M<br>HC=54% M	SCZ=46.8 (9.2)<br>HC=39.4 (9.1)	EDA	- MATRICS Consensus Cognitive Battery (MCCB) (including social cognition index)	- Patients as a whole performed significantly worse than HC's on almost all measures of neurocognition, social cognition and social function<br>- There were no differences observed between EDA-responding and non-responding patients in neurocognition or on most measures of social cognition and function.<br>- Patients had substantial deficits compared to healthy controls.

SCZ = Schizophrenia, **FDR =** First Degree Relatives, **HC** = Healthy Controls, **LF** = Low frequency power, **HF** = High frequency power, **SDNN** = Standard Deviation of NN Intervals, **SNS=** Sympathetic Nervous System, **PSNS =** Parasympathetic Nervous System, **ANS =** Autonomic Nervous System, **EDA =** Electrodermal Activity

Supplementary Table 3: Summary of studies assessing autonomic dysfunction in relation to metabolic dysfunction in SCZ

Author & Year	N	Sex (% Male)	Mean Age (SD)	HRV Measures	Metabolic Parameters	Significant Outcomes
Lee et al (2011)	SCZ=308<br>HC=719	SCZ:67%<br>HC: 71%	NR	HF<br>LF<br>LF/HF<br>SDNN	Metabolic Syndrome<br>Abdominal obesity<br>Triglycerides<br>HDL-C<br>Blood Pressure<br>Fasting Glucose	- SCZ patients have significantly lower cardiac autonomic control, however higher LF and HF values than healthy adults<br>- No significant differences between the SCZ patients with and without metabolic syndrome
Chung et al (2013)	SCZ=94<br>HC=51	SCZ:88%<br>HC:82%	SCZ:40.8 (8.9)<br>HC: 41.1 (9.1)	SDNN<br>RMSSD<br>pNN50<br>LF<br>HF<br>VLF	Wait circumference<br>BMI<br>Triglycerides<br>HDL-C<br>LDL-C<br>SBP<br>DBP<br>Fasting glucose	- Decreased HRV in patient groups compared to controls<br>- Reduced vagal HRV indices correlated with increased BMI, DBP, triglycerides, HDL and LDL.
Musa et al* (2016)	SCZ=45<br>SCZ+M=45<br>HC=45	NA	NA	Time domain HRV measures	NA	- SCZ patients at risk of metabolic syndrome displayed a reduced HRV compared to HC

SCZ = Schizophrenia, **HC** = Healthy Controls, **MS =** Metabolic Syndrome, **NA =** Not available to report on, **LF** = Low frequency power, **HF** = High frequency power, **VLF =** Very Low Frequency, **SDNN** = Standard Deviation of NN Intervals, **RMSSD** = Root Mean Square of the Successive Differences, **pNN50** = Mean number of times an hour in which the change in successive normal sinus (NN) intervals exceeds 50 ms, **BMI**= Body Mass Index, **DBP =** Diastolic Blood Pressure, **SBP=** Systolic Blood Pressure, **HDL-C =** High Density Lipoprotein Cholesterol, **LDL-C =** Low Density Lipoprotein Cholesterol
* Limited information to report on as only abstract of this study was available
